# New quantitative approaches reveal the spatial preference of nuclear compartments in mammalian fibroblasts

**DOI:** 10.1098/rsif.2014.0894

**Published:** 2015-03-06

**Authors:** David J. Weston, Richard A. Russell, Elizabeth Batty, Kirsten Jensen, David A. Stephens, Niall M. Adams, Paul S. Freemont

**Affiliations:** 1 Department of Computer Science and Information Systems, Birkbeck College, University of London, London, UK; 2 Department of Optometry and Visual Science, City University London, London, UK; 3 Section of Structural Biology, Department of Medicine, Imperial College London, South Kensington, London, UK; 4 Department of Mathematics, Imperial College London, South Kensington, London, UK; 5 Heilbronn Institute for Mathematical Research, University of Bristol, Bristol, UK; 6 Department of Mathematics and Statistics, McGill University, Montreal, Québec, Canada

**Keywords:** spatial point pattern analysis, nuclear compartments, spatial preference, thin-plate spline, intensity estimation, mammalian fibroblasts

## Abstract

The nuclei of higher eukaryotic cells display compartmentalization and certain nuclear compartments have been shown to follow a degree of spatial organization. To date, the study of nuclear organization has often involved simple quantitative procedures that struggle with both the irregularity of the nuclear boundary and the problem of handling replicate images. Such studies typically focus on inter-object distance, rather than spatial location within the nucleus. The concern of this paper is the spatial preference of nuclear compartments, for which we have developed statistical tools to quantitatively study and explore nuclear organization. These tools combine replicate images to generate ‘aggregate maps' which represent the spatial preferences of nuclear compartments. We present two examples of different compartments in mammalian fibroblasts (WI-38 and MRC-5) that demonstrate new knowledge of spatial preference within the cell nucleus. Specifically, the spatial preference of RNA polymerase II is preserved across normal and immortalized cells, whereas PML nuclear bodies exhibit a change in spatial preference from avoiding the centre in normal cells to exhibiting a preference for the centre in immortalized cells. In addition, we show that SC35 splicing speckles are excluded from the nuclear boundary and localize throughout the nucleoplasm and in the interchromatin space in non-transformed WI-38 cells. This new methodology is thus able to reveal the effect of large-scale perturbation on spatial architecture and preferences that would not be obvious from single cell imaging.

## 1. Introduction

The eukaryotic cell nucleus does not contain membrane-bound compartments yet shows a high degree of internal organization [1,2]. However, the principles that determine and regulate such organization—the locational preferences of compartments within the nucleus—are mainly unknown. A complicating factor is that nuclear architecture is dynamic [2,3] and can undergo reorganization during cell differentiation, proliferation and tumorigenesis [4], although many of the emerging models suggest self-organization as a mechanism for functional organization [5–7]. Several studies have shown that a number of nuclear bodies (NBs) can be formed through self-association and some have preferred spatial associations with specific gene loci [6,8]. However, it is still unclear whether the functional compartments have common spatial preferences within the nuclei and also whether such preferences are cell-type or cell-state specific.

In order to characterize any spatial organization found within the cell nucleus, extensive replicate data are required. However, with multiple images, the task of revealing spatial organization can yield ambiguous results when the images are analysed individually. Furthermore, current methods for information aggregation across images are prone to the high variability inherent to biological processes. Statements about the global organization of the nucleus are difficult because the nucleus contains no easily identifiable ‘landmarks' that allow nuclei to be transformed into a common coordinate system. The geometric centre of the nucleus is often used as a landmark from which the location of nuclear compartments can be calculated using ‘radial analysis' [9]. Usually, location is expressed as the distance between the centroid of the compartment and the nuclear centre, crudely normalized to remove variation in nuclear size but *not* shape. However, the centre of the nucleus has little functional relevance [9]. Furthermore, radial analysis offers a very limited description of spatial preference based on the distance(s) and angle(s) between compartments and the nuclear centroid. A different approach entails an exploratory spatial hypothesis test to determine if the observed pattern is consistent with the simplest spatial model: complete spatial randomness (CSR). However, we have previously shown that standard spatial statistics tools can miss underlying spatial structures, especially when the intensity of the signal (i.e. the number of nuclear compartments) is low [10].

Spatial nuclear organization and its association with functional organization is not well understood. This is due in part to the shortcomings of extant methodology. Thus, we developed computational and statistical tools to better characterize the spatial preferences of functional compartments in cell nuclei imaged using indirect immunofluorescence and confocal microscopy. These tools are designed to analyse compartments that are point-like and hence are amenable to spatial point pattern analysis. Using shape analysis and image registration methods (e.g. [11,12]), to aggregate spatial information across replicate images, we construct an atlas of these spatial preferences which we term an ‘aggregate map’ (AM). The AM can be thought of as a consensus representation of the nucleus and its functional components that has been computed from replicate images. The AM approach offers the opportunity to uncover the three-dimensional spatial organization of nuclear compartments which cannot be established from analysis of individual images.

Many standard techniques attempt to assess the spatial locations of a compartment of interest through its location relative to other compartments which themselves have an unknown spatial preference. This can mask the global spatial preference of the compartment of interest. New technologies such as 3C, 4C, 5C and Hi-C have been developed to capture chromosome conformation and further enable the analysis of nuclear organization [13,14]. These technologies facilitate the mapping of chromatin interactions not through direct visualization but indirectly via biochemical proximity and subsequent modelling and have contributed to an improved understanding of genome structure. In addition, the need for computational models to help analyse the large amounts of experimental data generated is very clear; these models allow full advantage to be taken of the powerful insight that conformation capture methods can provide into the architecture and organization of the nucleus [15]. The AM approach is directly focused on global spatial preferences. Implicit in our analysis of replicate images using AMs is the premise that any aspects of global nuclear organization, as opposed to localized and specific dynamic events, are present throughout a collection of cell nuclei [16].

**Figure 1.**
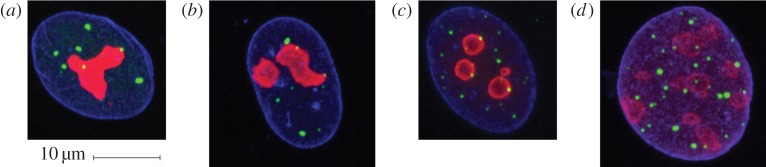
Functional compartments of the nucleus of (*a*) MRC-5 and (*b*) WI-38 fibroblasts, (*c*) SV40-transformed MRC-5 cells and (*d*) SV40-transformed WI-38 cells. Nuclei were imaged using indirect immunofluorescence and confocal microscopy in MRC-5 and WI-38 human diploid fibroblasts, and their SV40 T antigen-transformed MRC-5 and WI-38 counterparts. PML protein, which localizes to PML nuclear bodies (PML NBs), was also stained via indirect immunofluorescence in all images (green). The nuclear volume was delineated using indirect immunofluorescence directed against lamin B (blue), a protein of the nuclear lamina. Finally, nucleoli (red) have been imaged but are not the focus of this paper due to their extent which makes modelling their location by a point pattern problematic. These images are two-dimensional projections generated from the image data. The full three-dimensional data consists of image stacks, where each nucleus is represented by approximately 20 sequential slices of either 250 × 250 or 300 × 300 pixels. More details regarding the imaging can be found in electronic supplementary material, note 1.

Experiments were performed on the MRC-5 and WI-38 cell lines in a variety of conditions. Figure 1 shows examples of cell nuclei images in two-dimensional (2D) projection. It is clear from this figure that cell nuclei exhibit a large variation in boundary shape which complicates the spatial point pattern analysis. In addition, extra care is needed to reason about two-dimensional projections of three-dimensional objects. The AM methodology is designed to address both these issues.

## 2. Results

### 2.1. Construction of aggregate maps: image segmentation

**Figure 2.**
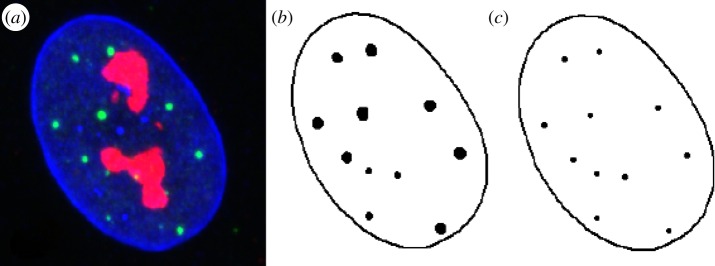
Two-dimensional illustration of the extraction of PML NB locations from image data. (*a*) Original raw image. (*b*) The boundary of the nuclear envelope and the PML NBs are segmented from an image (blue and green channels, respectively). The red channel represents nucleoli that are ignored in this analysis. (*c*) Each PML NB is replaced by its centre of gravity. Adapted from [10].

We begin with an RGB confocal microscopy *z*-stack image of a suitably prepared and selected cell nucleus, such as those in figure 1. The first step is to extract objects of interest from each image. Central to the AM methodology is the nuclear envelope, delineated by staining the protein, lamin B, in the blue channel (figure 1). Nuclear boundary voxels are segmented using the SCT [17] thresholding algorithm (although manual thresholding or other suitable segmentation algorithms could be used), followed by the construction of the convex hull. Informally, the convex hull of a set of points is a boundary that envelops all the points as tightly as possible and which has no indentations. The convex hull delineation is justified by the regular (smooth and convex) shape of MRC-5 and WI-38 fibroblast nuclei. Next, we identify compartment voxels. In figure 2*a*, the green channel refers to PML NBs which are selected by global thresholding using the SCT algorithm [17]. To facilitate the spatial point pattern analysis, we replace each compartment with its centre of gravity.

### 2.2. Construction of aggregate maps: identification of nuclear landmarks and computation of average nucleus

**Figure 3.**
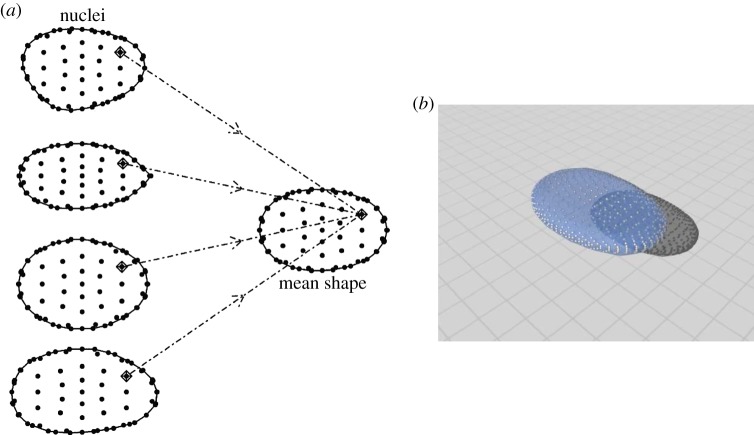
(*a*) Two-dimensional illustration of the landmark locations on cell nuclei. Landmarks are placed at regular intervals over the entire boundary in order to capture its shape. Correspondence between landmarks allows for the construction of a mean shape. Note that in this image, the landmarks are two-dimensional projections from a three-dimensional shape. (*b*) The three-dimensional mean shape derived from 50 nuclei from a normal, asynchronous MRC-5 dataset. This image also includes the landmark locations.

**Figure 4.**
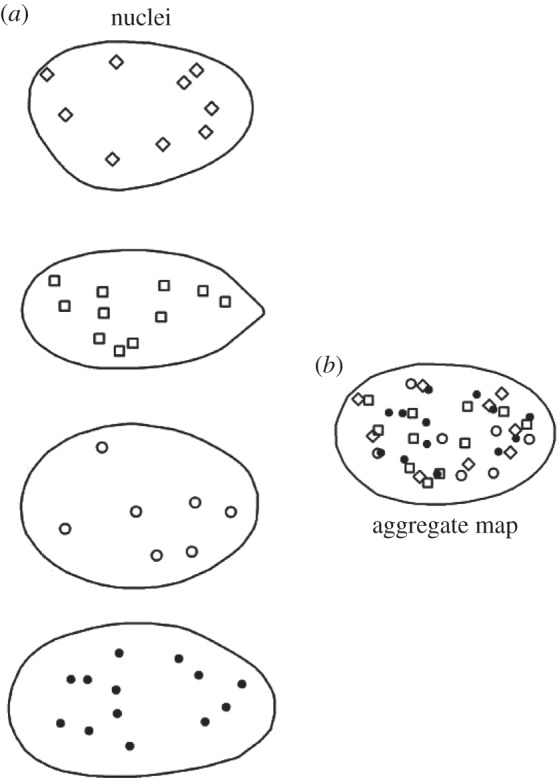
Two-dimensional illustration mapping compartments from cell nuclei (*a*) into a common frame of reference (*b*). This common frame of reference is based on the mean shape of the cell nuclei. The four nuclei shown in (*a*) have differing shapes and cannot be compared on an equal footing, or simply overlaid, as the nuclei are quite different (even in two dimensions). The compartment of interest from each cell has been projected into the mean shape and collectively these constitute the aggregate map (AM).

Having segmented a set of images, the next objective is to compute an average nuclear boundary to serve as the basis of the AM. We primarily employ standard techniques from statistical shape analysis [11,12] to construct this average boundary. However, we require landmarks—points of correspondence—on each nuclear envelope to calculate the average boundary. As noted in the Introduction, nuclei have no common biologically meaningful landmarks. To resolve this, we use the shape of the boundary to determine an ‘ovoid tip’, which is the location of the most pointed end of the nuclear boundary under two-dimensional projection. The nuclei shown in figures 3*a* and 4*a* are all rotated such that the so-called ovoid tip is rightmost on each nuclear boundary. The location of this tip and the direction of gravity are used to orient the nuclei (see electronic supplementary material, note 2). In order to capture the shape of the nuclear boundary, we add regularly spaced landmarks on the boundary starting from the ovoid tip, figure 3 shows an example of the result of this process. Note that this marking is performed in three dimensions, as discussed in more detail in electronic supplementary material, note 2. At this stage, an image has been reduced to a set of landmarks on the boundary and the interior locations of the compartments.

The critical aspect is that landmarks correspond across all replicate images. Having identified these corresponding landmarks, we are in a position to compute the average nuclear boundary which is computed using a statistical shape analysis procedure known as generalized procrustes analysis (GPA) [11]. The method selects parameters for shifts, rotations and scalings (the Euclidean similarity transforms), to minimize a sum of squares criterion, subject to a constraint on scale [11]. GPA yields a set of average landmarks associated with the minimizing solution, which characterize the average shape of all nuclei in the experiment. This process is illustrated in figure 3*a* and a real example of a mean shape is shown in figure 3*b*.

### 2.3. Construction of aggregate maps: image registration and intensity estimation

The next stage of our methodology transforms each processed image into the average boundary, as represented by the average landmarks. This requires a nonlinear spatial transformation (known as a deformation) of the landmarks from each nucleus to match the landmarks of the average shape. This crucial ‘registration’ stage places all the compartments of interest from each replicate into a common frame of reference (figure 4).

For each processed nucleus (the source), we transform its landmarks to those of the average nucleus (the target) using thin-plate splines [11,12]. This transformation places the source landmarks in exact correspondence with the associated target landmarks using rigid and non-rigid deformations. Naturally, the use of a non-rigid deformation may raise concerns. Such concerns are addressed in electronic supplementary material, note 4.

**Figure 5.**
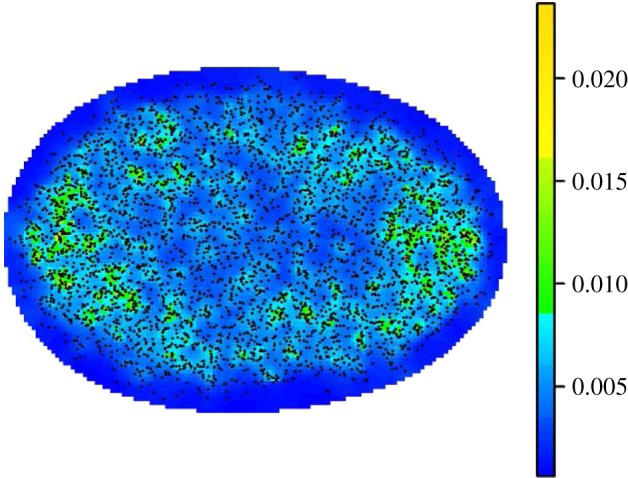
Two-dimensional orthogonal projection of PML NB centres and intensity estimates from normal, asynchronous MRC-5 cell nuclei. The dots represent the centres of all PML NBs mapped to the mean shape. The colour bar represents the estimate for the expected number of centres per unit volume (the intensity estimate). For this two-dimensional representation, the locations are orthogonally projected and we show the mean intensity estimate along the line of projection.

For convex objects, such as the nuclei we study, this mapping will always transform interior source voxels to the interior of the average boundary. The next step is ‘image fusion’, in which transformed nuclear compartments from all nuclei are combined. This yields a set of data which we refer to as an AM, that is, boundary landmarks, and compartments of interest transformed into the average boundary. Visualizing AMs requires further processing as there is usually significant over-plotting of compartment voxels, due to the potentially high number of images registered. A useful representation of the AM involves estimating the *intensity*, informally defined as the expected number of compartment centres at any given location. It is at this stage, we voxelize the AM: this is a common practice in brain mapping [18] and simplifies the process of intensity estimation which entails spatial smoothing of the observed point pattern. Three-dimensional spatial intensity estimation is difficult as methods frequently require the determination of a so-called bandwidth parameter that controls the degree of smoothing. To avoid the computationally demanding effort of determining such a parameter, we have developed our own approach called the *natural neighbour intensity estimation* algorithm which is described in detail in electronic supplementary material, note 3. For example, figure 5 shows the intensity estimate for the MRC-5 asynchronous AM, with the locations of PML NB centres overlaid.

**Figure 6.**
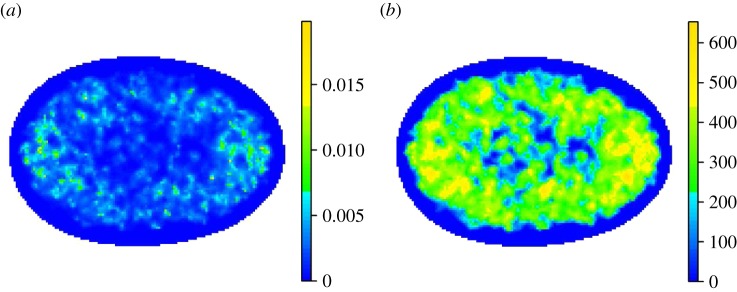
(*a*) Two-dimensional orthogonal projection of intensity above complete spatial randomness (CSR) for normal, asynchronous MRC-5 cell nuclei. At each voxel location within the boundary, the expected number of PML NB centres per unit volume is estimated. The expected number of PML NB centres per unit volume under CSR is subtracted with any values less than zero set to zero. The colour scale represents the mean of these values along the line of projection. This figure is dominated by peaks in the intensity estimate. (*b*) Corresponding result after a cluster enhancement step [16] has been introduced, details for which can be found in electronic supplementary material, note 3. The colour scale represents cluster size. The result is much smoother, in addition a hypothesis test based on the entire image (rather than on individual voxel locations) can be usefully constructed using these cluster values (see electronic supplementary material, note 3).

In order to assess the significance or otherwise in the fluctuations in intensity over the AM, we perform a hypothesis test, where the null hypothesis is that the true intensity function from each replicate corresponds to CSR. Testing all voxels simultaneously, naturally leads to issues regarding multiple hypothesis testing, therefore we adopt an approach called threshold-free cluster enhancement [16]. This involves measuring the size of connected regions of high or low intensity and calibrating against CSR using a Monte Carlo procedure. Further details for this approach can be found in electronic supplementary material, note 3. Figure 6 shows the intensity values above CSR and the corresponding cluster sizes.

### 2.4. Aggregate map results

**Figure 7.**
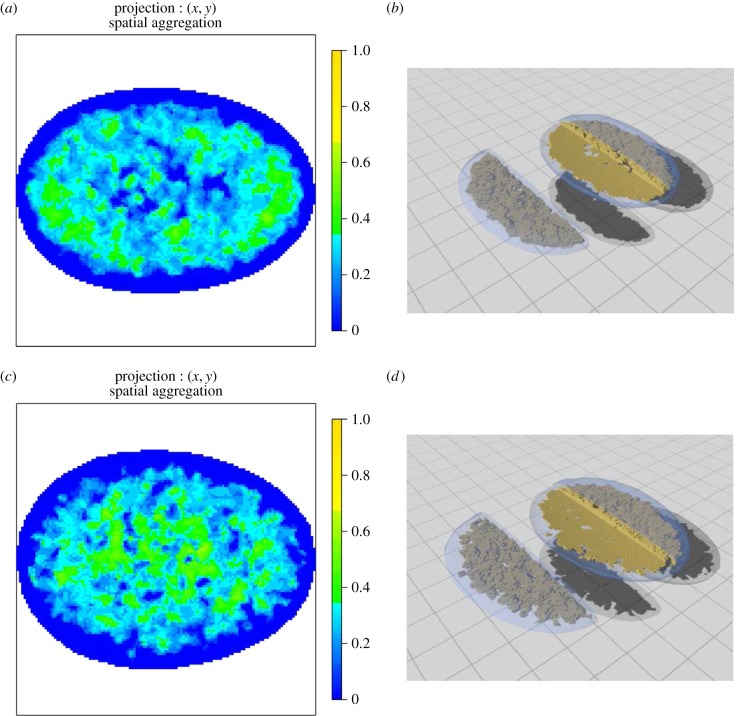
(*a*) The AM for the spatial preference of PML NBs in normal, asynchronous MRC-5 cells. The colour scale represents the probability that a voxel along the line of projection rejects the null hypothesis of CSR due to the PML NB centres being more aggregated than expected under CSR. (*b*) Full three-dimensional representation where each voxel that rejects the null is displayed. A segment from the nucleus has been removed to enable the centre of the map to be visualized. (*c*) The AM for SV40-transformed asynchronous MRC-5 cells and (*d*) the corresponding three-dimensional representation. Note the preference (*a*,*c*) for the centre in the SV40-transformed cells in contrast to the normal non-transformed MRC-5 cells.

There are various ways to represent the AM, for example figure 7 shows two-dimensional orthogonal projections and their corresponding full three-dimensional representation. It is useful to show an AM showing regions that are more aggregated than is expected under CSR and another AM showing regions that are more dispersed than expected under CSR. It should be noted that boundary effects can reduce the power of our approach. Specifically, regions near the boundary may fail to reject the null hypothesis in the presence of an alternative (see for example figure 12 and the discussion in electronic supplementary material, note 4).

The AMs shown in the following figures are two-dimensional orthogonal projections of three-dimensional AMs. For each figure, the colour represents the proportion of voxels that reject the null hypothesis of CSR along the line of projection (along the *z*-axis). The first column shows regions that are more aggregated than would be expected under CSR and the second column shows regions that are more dispersed than would be expected under CSR.

The colour scheme for the two-dimensional projections of three-dimensional AMs ranges from darkest yellow, which corresponds to all voxels along the line of projection rejecting the null hypothesis, to darkest blue which represents the case where no voxels reject the null. This two-dimensional representation of the three-dimensional AM is straightforward to interpret and allows all AMs to be displayed on the same colour scale. However, displaying the proportion of voxels that reject the null hypothesis can lead to a visual artefact especially close to the edge of the two-dimensional image. The *total* number of voxels along a line of projection varies across the AM and hence the volume a particular colour corresponds to varies across the image. Close to the edge of a two-dimensional AM, the number of voxels can be very small so it is possible to visually overestimate the size of the region that rejects the null given the colour.

**Figure 8.**
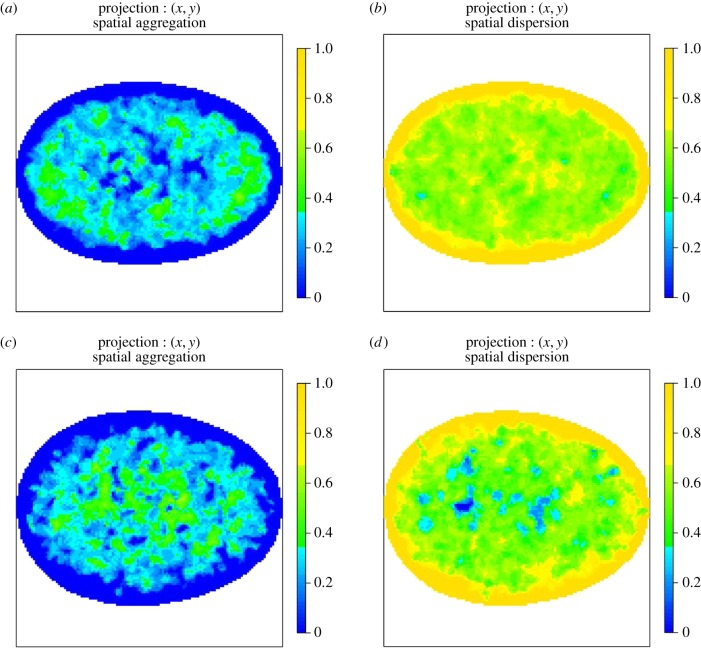
(*a*,*b*) AMs for the spatial preference of PML NBs in normal, asynchronous MRC-5 cells. The colour scale represents the probability that a voxel along the line of projection rejects the null hypothesis of CSR. In particular, panel (*a*) shows regions that reject the null due to the spatial preference being more aggregated than expected under CSR and panel (*b*) shows regions that reject the null due to the spatial preference being more dispersed than expected under CSR. In all cases, the colour blue denotes consistency with CSR. (*c*,*d*) AMs for the spatial preference of PML NBs in SV40-transformed asynchronous MRC-5 cells. Note the preference for the centre in the SV40-transformed cells in contrast to normal non-transformed MRC-5 cells.

**Figure 9.**
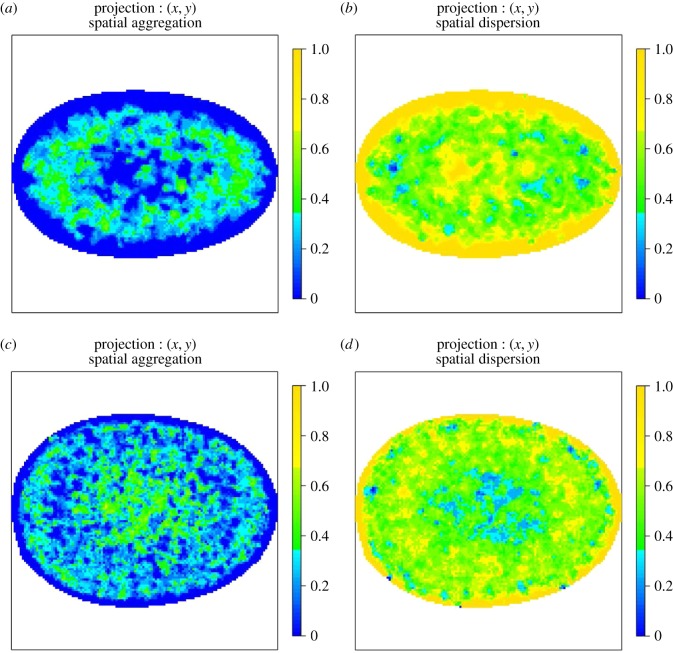
(*a*,*b*) AMs for the spatial preference of PML NBs in normal, asynchronous WI-38 cells and (*c*,*d*) SV40-transformed asynchronous WI-38 cells. The colour scale represents the probability that a voxel along the line of projection rejects the null hypothesis of CSR. (*a*,*c*) Regions that reject the null due to the spatial preference being more aggregated than expected under CSR and (*b*,*d*) regions that reject the null due to the spatial preference being more dispersed than expected under CSR. In all cases, the colour blue denotes consistency with CSR. Note the preference for the centre in the SV40-transformed cells in contrast to normal non-transformed cells. This is a similar to the behaviour of PML NBs in MRC-5 cells.

Figure 8 shows the AM for PML NBs in normal, asynchronous MRC-5 cells and SV40-transformed asynchronous MRC-5 cells. Notably, there is a preference in the normal non-transformed cells which is mainly annular with polar preferences. A similar pattern of behaviour can be seen using the WI-38 cell line (figure 9).

**Figure 10.**
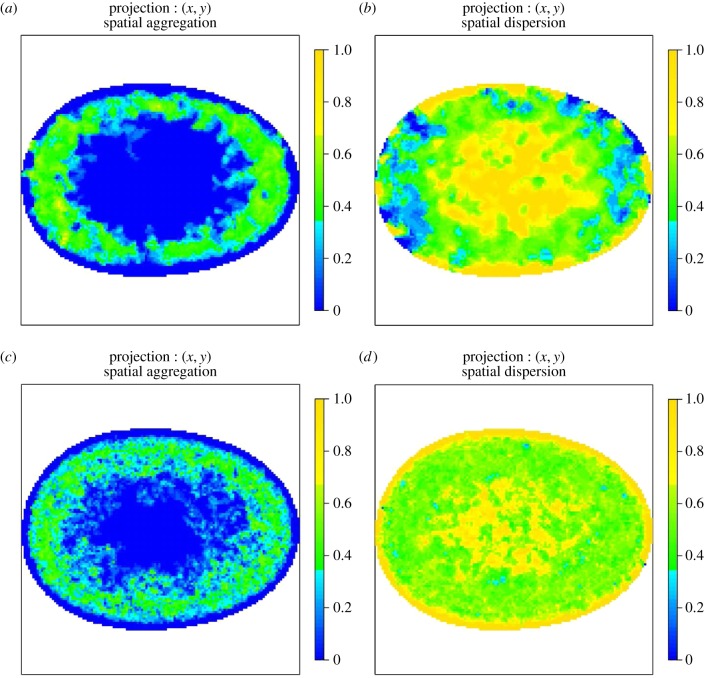
(*a*,*b*) AMs for the spatial preference of RNAP II in normal, asynchronous MRC-5 cells and (*c*,*d*) SV40-transformed asynchronous MRC-5 cells. The colour scale represents the probability that a voxel along the line of projection rejects the null hypothesis of CSR. (*a*,*c*) Regions that reject the null due to the spatial preference being more aggregated than expected under CSR and (*b*,*d*) regions that reject the null due to the spatial preference being more dispersed than expected under CSR. In all cases, the colour blue denotes consistency with CSR. The spatial preference of RNAP II is mapped for both normal, asynchronous MRC-5 cells (top row) and SV40-transformed asynchronous MRC-5 cells (bottom row) and is shown to be preserved upon SV40 transformation.

**Figure 11.**
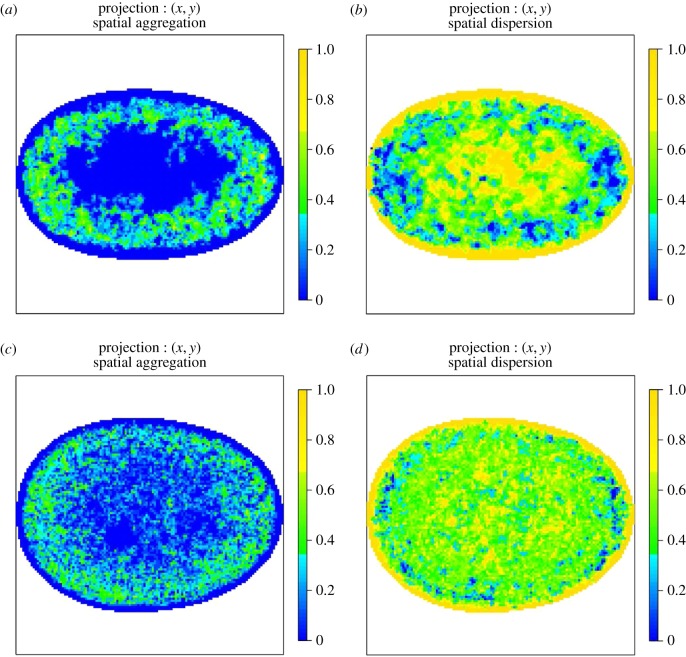
(*a*,*b*) AMs for the spatial preference of RNAP II in normal, asynchronous WI-38 cells and (*c*,*d*) SV40-transformed asynchronous WI-38 cells. The colour scale represents the probability that a voxel along the line of projection rejects the null hypothesis of CSR. (*a*,*c*) Regions that reject the null due to the spatial preference being more aggregated than expected under CSR and (*b*,*d*) regions that reject the null due to the spatial preference being more dispersed than expected under CSR. In all cases, the colour blue denotes consistency with CSR. The spatial preference of RNAP II is mapped for both normal, asynchronous WI-38 cells (top row) and SV40-transformed asynchronous WI-38 cells (bottom row) and is shown to be preserved upon SV40 transformation.

Figures 10 and 11 show corresponding AMs for RNA polymerase II (RNAP II). Strikingly, the spatial regularity of RNAP II is preserved between normal, asynchronous cells and their SV40-transformed counterparts and reveals an annular preference surrounding the nuclear envelope. This suggests that RNAP II spatial regularity in the nucleus must be preserved even when a cell undergoes immortalization, such as upon transformation with the SV40 antigen and that high-levels of transcriptional activity may occur near the nuclear periphery but not at the nuclear envelope. Given the resolution of our analyses, our observations are consistent with transcription occurring within the perichromatin region [19]. Our results do however indicate a preference for transcriptional activity that is excluded from the central part of the nucleus. It is important to note that our AM maps show spatial propensity of the imaged compartments and thus do not exclude compartments occuring anywhere within the nucleus.

There were no major differences between the AM patterns for all of the nuclear compartments we studied in the SV40-transformed MRC-5 and the SV40-transformed WI-38 cell lines. Overall, this suggests that the higher order organization of transformed cells is conserved across similar cell types (fibroblasts). Despite our observations that SV40 transformation appears to lead to significant changes in higher order organization when compared to the organization of normal, non-transformed nuclei, the effects of such a transformation are similar across alternative transformed cell lines. It is possible that cell cycle differences between normal and transformed cells could affect the patterns we observe [20], nevertheless, our observations are consistent for four different cell lines.

**Figure 12.**
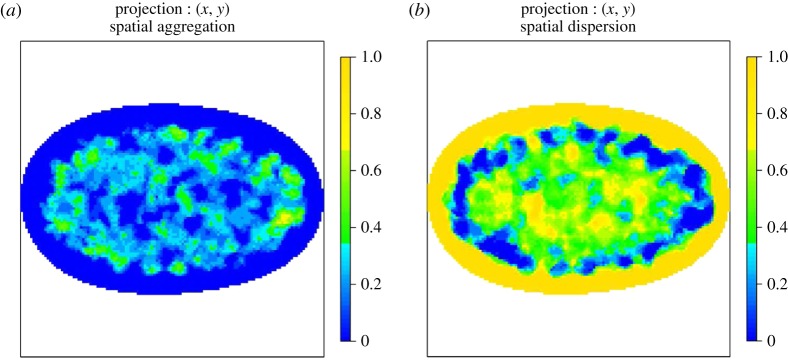
AMs for SC35 splicing speckles in normal, asynchronous WI-38 cells. The colour scale represents the probability that a voxel along the line of projection rejects the null hypothesis of CSR. (*a*) Regions that reject the null due to the spatial preference being more aggregated than expected under CSR and (*b*) regions that reject the null due to the spatial preference being more dispersed than expected under CSR. In all cases, the colour blue denotes consistency with CSR. In both cases, blue denotes consistency with CSR. Note that SC35 splicing speckles are excluded from the nuclear boundary consistent with localization throughout the nucleoplasm and interchromatin space.

Finally, figure 12 shows the AM for SC35 splicing speckles in normal, asynchronous WI-38 nuclei. This AM confirms that SC35 splicing speckles are excluded from the nuclear boundary and appear to be localized throughout the nucleoplasm and in the interchromatin space [21].

## 3. Discussion

Image processing and subsequent analysis is hugely important for cell biology. The amount of image data in this field is growing rapidly, driven by advances in microscopy technology (e.g. high-throughput microscopy and increased resolution). As a result, there is an increasing need to replace qualitative visual assessment and manual measurements of microscope images with quantitative automated image analysis methods, especially for replicate images.

Here, we present a new technique to explore spatial preference within the cell nucleus using biological confocal microscopy image data. The strength of this methodology derives from the principled way replicates are combined where the shape of each nuclear boundary is respected. This necessitates the analysis to be performed in full three dimensions.

Our approach to combining replicates involves a nonlinear projection which can produce artefacts in the AM, however we have shown through simulation that these artefacts are small and localized. Furthermore, the effect of artefacts is mitigated through our method for making a formal inference decision regarding a null hypothesis of CSR. In our approach, the null distribution is constructed by generating CSR replicates that are then projected into the AM. In other words, the AMs drawn from the null distribution are generated from CSR replicates that have themselves been similarly nonlinearly transformed.

Central to the AM construction is the ability to find correspondences between replicates, for this we use the location of an ovoid tip that is determined automatically. This does not preclude manual intervention in cases where the presence of an ovoid tip is not strongly evident. Other than this scenario, the construction of an AM requires little to no input from the user. There are two main parameters that the user might wish to tune. First, the number of landmarks used to represent the convex hull of the nuclear boundary, this is unlikely to need any fine tuning due to the relative simplicity of this shape. Second, for the intensity estimation, the contents of the AM are discretized, a very small voxel size relative to the size of the AM is unlikely to add any further relevant detail to the AM (but will substantially increase the computation) hence it unlikely that there will be a need to change the default number of voxels used in all the results shown in this paper.

By applying the AM methodology to visualize a number of nuclear compartments including PML NBs and RNAP II, we have identified interesting spatial preferences that have not been observed previously. These observations provide a framework to explore further nuclear compartments and cellular perturbations to define high-level relationships between spatial preference and cellular function. Much of the previous work on nuclear compartment associations has been carried out using single-cell imaging or biochemical cross-linking techniques. Both approaches have revealed significant information about nuclear organization but can potentially mask meso-level spatial preferences. In an attempt to provide a more global view of nuclear organization, [22] developed a computational probability approach to analyse nuclear organization in budding yeast and found distinct association preferences. Here, we have developed a different technique and applied it to mammalian interphase nuclei, and similar to [22] we also observe distinct preferences and associations. A full description of these associations will be published elsewhere.

The AM and other such tools are becoming essential as they reduce substantial manual labour and more importantly remove subjective bias. In addition, such quantitative methods can increase the accuracy, sensitivity and reproducibility of data analysis.

## 4. Material and methods

Further details on the cells investigated, including experimental methods for cell staining, indirect immunofluorescence and confocal microscopy can be found in electronic supplementary material, note 1. Briefly, we chose to analyse two primary human diploid cell lines, MRC-5 and WI-38 fetal lung fibroblasts, and their SV40 T-antigen-transformed counterparts in order to avoid complications with disease-derived cell lines. By using immunolabelling and confocal microscopy to collect *z*-stack images, we captured over 1000 image stacks of nuclei labelled for the nuclear envelope (lamin B protein), and the nuclear compartment known as PML NBs (see figure 1 for example images).

## Supplementary Material

Supplementary Material for: New quantitative approaches reveal the spatial preference of nuclear compartments in mammalian fibroblasts

## Data accessibility

The three-dimensional spatial locations of objects of interest used in the analysis and raw image data for figure 12 can be found at Dryad doi:10.5061/dryad.d3mr7.
