# Supplementary material for: New quantitative approaches reveal the spatial preference of nuclear compartments in mammalian fibroblasts
Source: J R Soc Interface. 2015 Mar 6;12(104):20140894. doi: 10.1098/rsif.2014.0894 (PMC4345468; doi:10.1098/rsif.2014.0894)
Supplement: Supplementary Material for: New quantitative approaches reveal the spatial preference of nuclear compartments in mammalian fibroblasts [file rsif20140894supp1.pdf]

Supplementary Material for: New quantitative approaches reveal the spatial preference of nuclear compartments in mammalian fibroblasts

David J. Weston[1,*], Richard A. Russell[2], Elizabeth Batty[3], Kirsten Jensen[3], David A. Stephens[6], Niall M. Adams[4,5,*], Paul S. Freemont[3,*],

1 Department of Computer Science and Information Systems, Birkbeck College, University of London, London, United Kingdom.
2 Department of Optometry and Visual Science, City University London, United Kingdom.
3 Macromolecular Structure and Function Laboratory, 4 Department of Mathematics, Imperial College London, South Kensington, London, United Kingdom.
5 Heilbronn Institute for Mathematical Research, University of Bristol, Bristol, United Kingdom.
6 Department of Mathematics and Statistics, McGill University, Canada.
∗ E-mail: dweston@dcs.bbk.ac.uk, n.adams@imperial.ac.uk, p.freemont@imperial.ac.uk.

Supplementary Note 1: Cell lines and immunolabeling

Cells were cultured in T25 or T75 flasks at 37°C and 5% CO_2. Cell lines and corresponding culture media used were as follows. MRC-5 human Caucasian male fetal lung fibroblasts, a normal diploid human cell line (ATCC) were cultured in RPMI 1640 supplemented with 10% FCS, 2mM L-glutamine, and Penstrep (50 IU/ml penicillin, 50μg/ml streptomycin), (all Invitrogen). WI-38 human Caucasian female fetal lung fibroblasts (ECACC) were cultured in MEM with Earles' Salts supplemented with 10% FCS, 2mM L-glutamine, 1% NEAA, 1mM sodium pyruvate and Penstrep (all Invitrogen). MRC-5 VA (MRC-5 SV2) SV40-transformed MRC-5 cells (obtained from ICRF) were cultured in DMEM supplemented with 10% FCS, 2mM L-glutamine, and Penstrep (all Invitrogen). SV40-transformed WI-38 cells (WI-38 VA13 Subline 2RA or WI-38 VA (ECACC)) were cultured in MEM with Earles Salts supplemented with 10% FCS, 2mM L-glutamine, 1% NEAA, 1mM sodium pyruvate, and Penstrep (all Invitrogen).

In order to determine if the spatial preferences determined for a compartment of interest in MRC-5 and their SV40-transformed counterparts were applicable to other human fibroblasts, the experiments were repeated in both the WI-38 fetal lung fibroblast and SV40-transformed WI-38 VA cell lines. Cells were plated onto glass coverslips in six well plates and incubated at 37C and 5% CO_2. After 48 hours cells were fixed in 4% paraformaldehyde and permeabilized in 0.5% Triton X-100. Cells were concurrently incubated with three primary antibodies against nuclear antigens for 30 min at 37°C. After washing they were incubated with appropriate secondary antibodies for 30 min at 37°C in the dark.

Immunolabeling and confocal microscopy

A mouse anti-PML antibody (Santa Cruz Biotechnology Inc. (PML (PG-M3): sc-966)) and a donkey anti-mouse Cy3 secondary antibody were employed to detect PML NBs. The edge of the nucleus was delineated with a goat anti-Lamin B antibody (Santa Cruz Biotechnology Inc. (Lamin B (M-20): sc-6217), and labeled with a donkey anti-goat Cy5 secondary antibody. Slides were mounted in 0.25μg/ml DAPI, which allowed nuclei to be located for imaging. Z-stacks of nuclei were captured using a Zeiss LSM 510 confocal microscope with a Zeiss Plan Apochromat 63 x oil immersion objective, numerical aperture of 1.4 and a zoom of 3.4. Z-stacks typically consisted of about 20 sequential slices of 250×250 or 300×300 pixels (where 1μm = 12pixels) captured at 0.4μm intervals through the nucleus.

Primary antibodies:

Compartment	Species	Specificity/concentration of antibody	Source	Titer
PML nuclear body	Mouse Monoclonal IgG1	All isoforms of mouse, rat and human PML. 200μg/ml.	Santa Cruz Biotechnology Inc. (PML (PG-M3): sc-966).	1 in 100
RNA polymerase II	Mouse Monoclonal IgM	Yeast and human. 2-3 mg/ml	BabCo MMS-129R (H5)	1 in 500
SC35, splicing factor SC-35	Mouse Monoclonal IgG1	Rat, newt, frog, human and *Drosophila*	Sigma-Aldrich S4045 (clone SC-35)	1 in 500
Lamin B	Goat IgG	Lamin B of mouse, rat and human. $0.2\mu g/\mu l$.	Santa Cruz Biotechnology Inc. (Lamin B (M-20): sc-6217)	1 in 100

Secondary antibodies:

Species	Specificity/concentration of antibody	Fluor conjugate	Source	Titer
Donkey	Mouse IgG, 1.4mg/ml	Cy3	Jackson ImmunoResearch Laboratories, Inc.	1 in 200
Donkey	Goat IgG, 1.5mg/ml	Cy5	Jackson ImmunoResearch Laboratories, Inc.	1 in 200

Supplementary Note: 2

In this note, we give more details on the steps of the construction of aggregate maps, in the order delineated in the main document. An exploration of the strengths and weaknesses of the methodology is given in Supplementary Note 4.

Landmarks

In order to combine spatial point patterns from a collection of cell nuclei, we first identify landmarks on each nucleus boundary. A landmark, using the definition from [1], is a point of correspondence on each object that matches between and within populations.

For the case of nuclear boundaries there does not appear to be an immediately obvious set of landmark locations. So, the following assumptions are used to facilitate determining correspondences across cell nuclei boundaries. First, we assume the direction of gravity is a preferred direction within the cell nuclei.

Second, we assume that the cell nuclei are ovoidal and that in a 2D orthogonal projection onto the x-y plane, the outlines of the cell nuclei are approximately ellipsoidal. In particular we assume the two extrema along the major axis can be determined and distinguished from each other by their curvature. We use the extrema with the highest curvature as the location of the landmark (strictly a mathematical landmark) in 2D. The landmark in 3D is the location of the point on the boundary that is orthogonally projected onto the 2D landmark.

What remains is to identify *pseudo-landmarks* [1]. These are regularly spaced locations that are intended to adequately represent the nuclear boundary. For this we first align the nuclei, using translation and rotation, such that the centre of gravity of the boundary is located at the origin and the landmark on the ovoidal tip is on the positive $x-$axis. The placement of pseudo-landmarks is inspired from texture mapping in computer graphics, where an image is mapped onto the surface of an object with as minimal distortion as possible. There are many approaches for this in the literature, however in our case the boundary shape is assumed to be contained within a simple convex hull and hence we deploy a method described in [2]. Pseudo-landmarks are first placed onto a simpler shaped object, in our case a sphere, and then projected onto the boundary. First equi-spaced pseudo-landmarks are placed onto the boundary of a sphere centred at the origin. This sphere is then linearly stretched such that its bounding box matches the bounding box of the cell nucleus. Each pseudo-landmark is then projected onto the boundary along the line joining that pseudo-landmark to the shape's centroid.

The default number of landmarks has been set to 642 which corresponds to vertices from an icosahedron that has its facets split recursively to a depth 3. These vertices are projected onto a sphere to produce the equi-spaced pseudo-landmarks.

Generalised Procrustes Analysis and Thin-Plate Splines

Consider two shapes A, B that have n corresponding landmarks $A_1 \dots A_n$ and $B_1 \dots B_n$. The Procrustes distance d_P is defined as

$$d_P(A, B) = \min \sum_i^n d_E(A_i, T(B_i))$$

where the Euclidean distance is d_E and T is the set of shape preserving transformations. In our case, this includes translation, rotation and scaling; but not reflection.

The *mean shape*, M consists of a set of n landmarks. The mean shape of a set of k shapes $S_1 \dots S_k$ is defined as having landmark locations $M_1 \dots M_n$ that minimizes

$$\sum_i^k d_P(M, S_i)$$

This optimization is performed by an iterative procedure [1].

Once the mean shape has been determined, locations of compartments within each registered cell nuclei can be mapped into the mean shape. It can be seen in Figure **??**A that the mapping to the mean shape requires a non-linear deformation. The thin-plate spline provides a continuous transformation in such a way that every landmark is precisely mapped to its corresponding landmark, [1, p. 205–209]. Any interior point is represented as the weighted sum of the landmarks. These weights are in part determined by the choice of *radial basis function*, which in three-dimensions is simply taken to be the Euclidean distance. Moreover the weights are determined using the constraint that corresponding landmarks must map to the same location. This yields a system of linear equations. The resulting transformation can be conveniently split into an affine transformation, that minimizes the Procrustes distance, plus a non-linear deformation.

Supplementary Note 3

Natural Neighbour intensity estimation

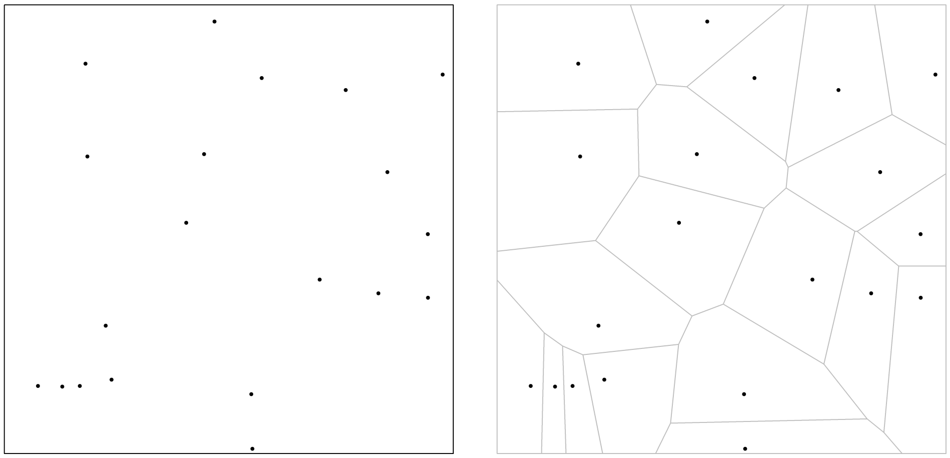

Figure 1: Constructing a 2D Voronoi graph. A) Spatial point pattern. B) The corresponding segmentation of the space due to Voronoi tessellation.

The method we developed to estimate the intensity function of a spatial point pattern, called Natural Neighbour intensity estimation (NNI), is both parameter free and adaptive. It makes use of a method for interpolating scattered data called natural neighbour interpolation. Before we explain the intensity estimation method in detail we describe this interpolation procedure.

The natural neighbours of an event in the spatial point pattern (or indeed any finite point set), is the set of neighbouring events from the Delaunay Triangulation of the spatial point pattern, see for example Figure 1 of [3]. If we assume that we have known intensities at each event e_i, the intensity estimate at the location x is simply a weighted sum of the intensities of the natural neighbours of x. Several weighting schemes have been proposed in the literature and we use the original scheme due to Sibson which can be conveniently optimized for discretized space [4]. The weights are calculated by first constructing the Voronoi graph of the original spatial point pattern, Figure 1. Next, an event at location x is added to the spatial point pattern in order to produce a second Voronoi graph, represented by a square in Figure 2. It can be seen, when overlaying these two graphs, that by introducing this extra event, regions that were once part of the Voronoi cells of the natural neighbor set of x are now part of the Voronoi cell of the event at x, Figure 3. The weight assigned to a natural neighbour of x is the size of the region taken from its original Voronoi cell and which is now part of the Voronoi cell of x. The weights are normalized such that the sum of these weights is one. More formally, if we assume we already have intensity estimate $\hat{\lambda}_i$ for each event e_i, we can use Natural Neighbour interpolation to estimate the intensity $\hat{\lambda}(x)$ at any location x,

$$\hat{\lambda}(x) = \frac{\sum_{i \in \mathrm{NN}(x)} w_{i,x} \hat{\lambda}_i}{\sum_{i \in \mathrm{NN}(x)} w_{i,x}}$$

where $\mathrm{NN}(x)$ are the natural neighbours of x. To simplify notation, we extend the summation over all

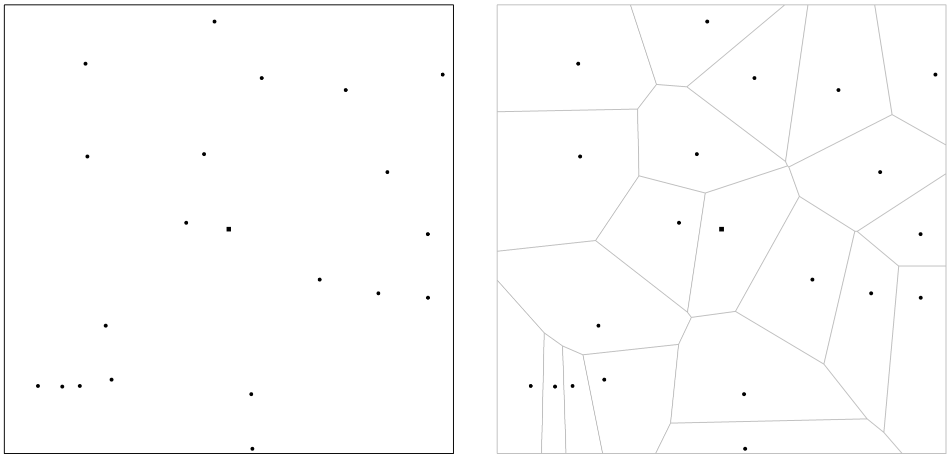

Figure 2: **Constructing a 2D Voronoi graph with an additional point.** A) The original point pattern from Figure 1 now with an extra point introduced displayed here as a square (located, for convenience, at the centre of the image). B) The Voronoi diagram of the spatial point pattern and the additional point.

events and set the weight to zero if the event is not a natural neighbor, i.e.

$$\hat{\lambda}(x) = \frac{\sum_{i=1}^{n} w_{i,x}\hat{\lambda}_i}{\sum_{i=1}^{n} w_{i,x}}$$

where $w_{i,x} = 0$ if $e_i \notin \mathrm{NN}(x)$. We consider the unknown intensity to be a scalar field $\lambda(\vec{r})$ in 2D or 3D.

We intend to use the above interpolation method to estimate the intensity of an inhomogeneous spatial point pattern. Furthermore we intend to ensure that the maximum likelihood estimate of the mean intensity of this pattern equals the mean of our intensity estimate, $\hat{\lambda}(x)$.

Let R denote the region of interest with size $|R|$, then the mean intensity over this region is

$$\frac{1}{|R|}\int_{|R|} \hat{\lambda}(\vec{r})d\vec{r}$$

The maximum likelihood estimate for the intensity given a spatial point pattern with n events in region R is simply

$$\frac{n}{|R|}.$$

Matching these two estimates, and simplifying gives,

$$\int_{|R|} \hat{\lambda}(\vec{r})d\vec{r} = n. \tag{1}$$

The actual contribution the intensity at event e_i makes to the intensity at a particular location x is

$$\frac{w_{i,x}\hat{\lambda}_i}{\sum_{j=1}^{n} w_{j,x}},$$

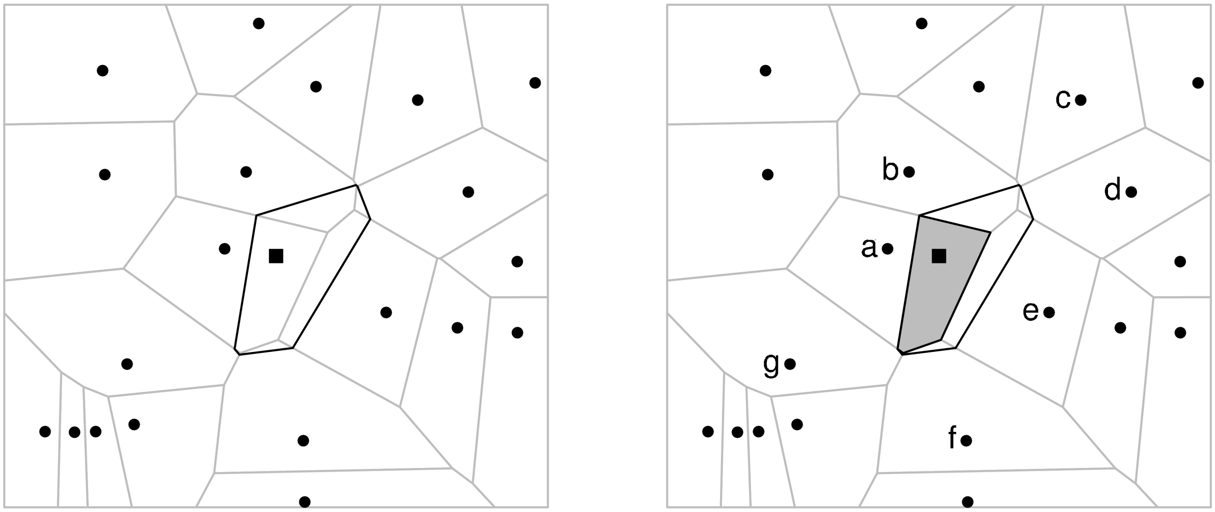

Figure 3: Overlaying the Voronoi tessellation with the additional point (Figure 2) on to the original the Voronoi tessellation (Figure 2). A) reveals the region that has now been associated with the new point. B) the region taken from one cell of the original tessellation (shaded region).

see Figure 3 for a graphical representation. The total contribution c_i of event e_i (see Figure 5) to the interpolated intensity over the entire discretized region of interest A,

$$c_i = \hat{\lambda}_i \sum_{x \in A} \frac{w_{i,x}}{\sum_{j=1}^{n} w_{j,x}}. \tag{2}$$

The sum of the intensity over the region is

$$\sum_{i=1}^{n} c_i$$

To satisfy our requirement that the maximum likelihood estimate of the mean intensity of this pattern equals the mean of our intensity estimate the sum must equal n, see Eq. 1,

$$\sum_{i=1}^{n} c_i = n \tag{3}$$

Substituting Eq. 1 into Eq. 3 yields,

$$n = \sum_{i=1}^{n} \left(\hat{\lambda}_i \sum_{x \in A} \frac{w_{i,x}}{\sum_{j=1}^{n} w_{j,x}} \right). \tag{4}$$

Eq. 4 constrains the values for $\hat{\lambda}_i$, one possible solution is to set the intensity estimates to,

$$\hat{\lambda}_i = \frac{1}{\sum_{x \in A} \frac{w_{i,x}}{\sum_{j=1}^{n} w_{j,x}}}.$$

This solution is the natural neighbour intensity estimate used with Aggregate Maps and has a simple geometric meaning. As discussed above, each event has a region it contributes to and this region contains

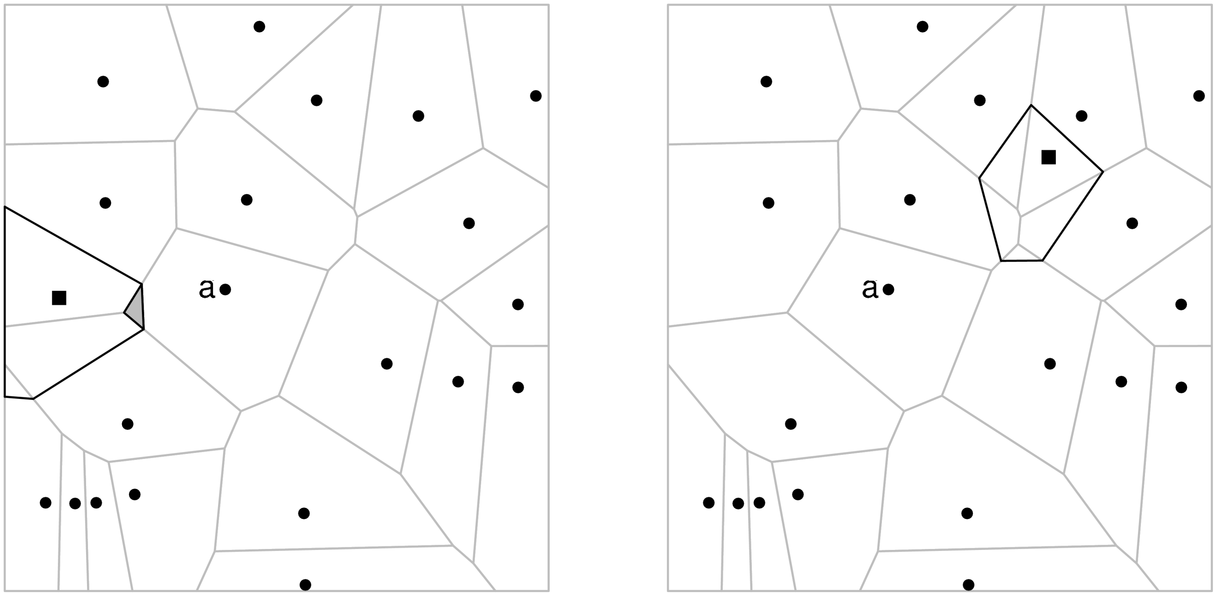

Figure 4: The contribution of e_i to 2 locations. A) The gray area represents the unnormalized contribution whereas in B) there is no contribution.

only one event. The intensity estimate we use is simply the number of events in this region divided by the (weighted) size of this region.

As has been previously mentioned, a version of natural neighbour interpolation based on discrete space has been used. The level of discretization has been set by setting the length of the size of each voxel to $128th$ the length of the longest length in the AM.

A problem with the Natural Neighbour Intensity Estimator, and with similar intensity estimation procedures based on tessellations such as DFTE [5], is that the intensity estimates have high variance [6]. In the next section we discuss hypothesis tests applied to the estimated intensity function and we describe an approach that mitigates issues regarding multiple hypothesis testing and smooths the high variance intensity estimates.

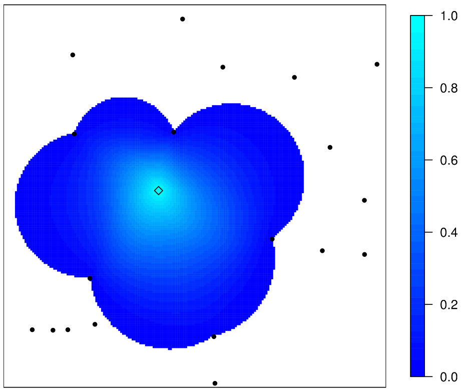

Figure 5: **The normalized contribution of e_i for each location within the square.** The highest contribution to the interpolated value occurs at the location of e_i, whereas outside the shaded region e_i does not contribute to the interpolated value.

Threshold Free Cluster Enhanced Inference (TFCE)

Deploying NNI on the Aggregate Map results in an intensity estimate for each voxel in the AM. In order to assess the significance or otherwise in the fluctuations in intensity over the AM we perform an hypothesis test, where the null hypothesis is that the true intensity function from each replicate corresponds to CSR. We deploy a simple non-parametric permutation test as follows.

First, for each original nucleus we create 100 samples of aggregate maps from the null. These samples respect the boundary of the original nucleus. This is achieved by generating events that follow CSR where the number of events is equal to the number of events in each replicate. These CSR events are then projected into the mean shape and the intensity estimated. This step is intended to mitigate the effects of the non-linear aspect of the thin-plate spline projection. Testing all voxels simultaneously naturally leads to issues regarding multiple hypothesis testing.

We address this problem by using the clustering approach described in [7]. This approach is illustrated for a 1D function. Each location x has a region associated with it $A(x)$, e.g. for point a in Figure 6A where $A(x)$ is the shaded region in Figure 6B.

The cluster value at a location x is defined as an integral over this region. The integrand is a function that has two has control parameters denoted e and h that are set to 0.5, 2 respectively. We adapt this approach by setting both $e = 1$ and $h = 0$. This reduces the integration to simply calculating the area under the intensity curve. The rationale here is to suppress the high variance in the intensity, where we lend more evidential weight to regions that are large but only slightly above CSR intensity, and less to regions that are small but have a high intensity. Figure 7 shows the cluster sizes corresponding to the intensities of Figure 6A.

The top row of Figure 8 show two versions of the intensities shown in Figure 7A but with additional spikes. The bottom row of Figure 8 shows their corresponding cluster values. These TFCE cluster values are robust to the spikes. Such spikes in the estimated intensity can occur with our natural neighbor intensity estimator since events can occur arbitrarily close to each other. Note we have only added spikes to the intensity not troughs in this one dimensional example. In one dimension troughs will have a large impact on the cluster size, but will have much less impact in two or higher dimensions (this is due to connectivity of the space). We are not concerned with using our AM approach to 1-D signals so this issue with TFCE cluster size does not impact on the AM methodology.

Measuring cluster sizes enables us to avoid the multiple hypothesis testing problem by recording, for

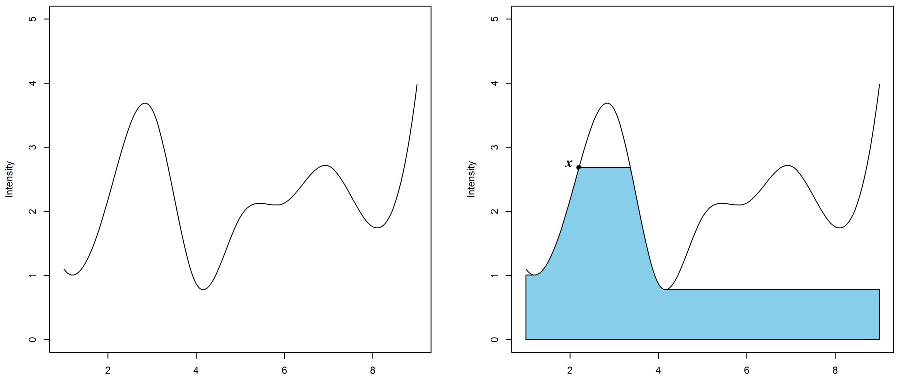

Figure 6: A) Intensity Estimate, B) The region that supports location x

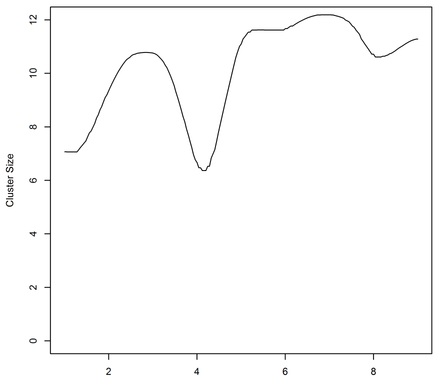

Figure 7: The TFCE graph corresponding to the intensity estimates from Figure 6A.

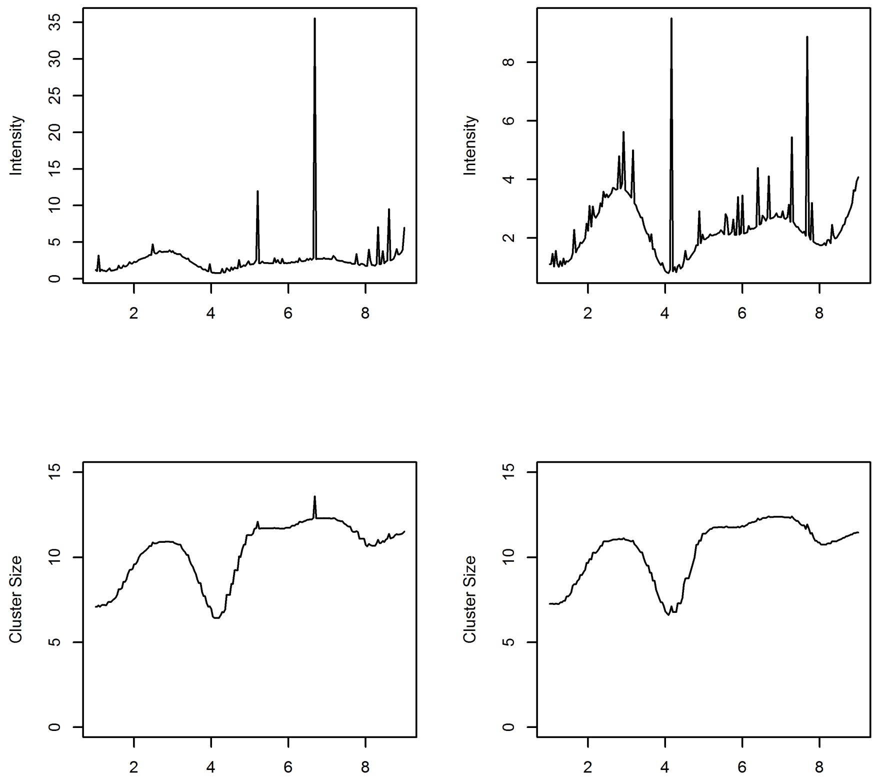

Figure 8: **TFCE graphs corresponding to intensity estimates with added intensity spikes (top row).** The bottom row shows the corresponding TFCE cluster sizes. Note how robust the TFCE graphs are to spikes in intensity.

each null replicate, the size of the largest cluster. The size of clusters found in the original map are calibrated using the p-value from the distribution of the null cluster sizes. A cluster size that is larger than a specified p-value threshold rejects the null hypothesis of CSR.

We are interested in regions that are more aggregated than is expected under CSR but also regions that are more dispersed than expected under CSR. Hence we need to perform a two-tailed test. In order to achieve this using the one-tailed test described above, we first subtract the mean of the intensity estimate (note, due to our method for estimating intensity each replicate and the target map all have the same mean) from each null replicate and the target image. The above one-tailed test is performed twice. For testing for regions that are more connected than CSR, we proceed to the next step; For testing regions that are more dispersed than CSR we multiply all intensity estimates by -1. Then any intensity estimates less than zero are set to zero and the above threshold free cluster size test is performed at the 97.5 percentile threshold.

Supplementary Note 4: AM Verification

A number of issues may be of concern with the steps in the AM methodology.

1. The effect of the non-linear deformation.
2. The impact of the ovoidal tip as a landmark.
3. The detection capability of the methodology.

The following sections address these issues.

Non-Linear Deformation

Since the TPS transformation includes a non-linear deformation, there may be concern that artefacts arise as a result of the transformation rather than being aspects of the underlying process. To explore the extent of the TPS transformation, Figure 9 illustrates the variability induced by the transformation for synthetic data (see next section for details of data construction). The key observation arises in the bottom row of the figure where we see that the spread of each individual point is localized to a relatively small volume within the boundary. Broadly speaking there is very little overlap between the points although we see that the deformation gets larger the further the point is from the centre.

Figure 10 shows corresponding results for real data, in this case normal, asynchronous MRC-5 fibroblasts. The variation in perturbation is small relative to the size of the nucleus. A way to quantify the perturbation is to measure the ratio of the volume of the 95% confidence contour to the volume of the shape boundary; see Figure 11. In both cases the volume ratio is very small (less than one in 500 for both the real and synthetic cases).

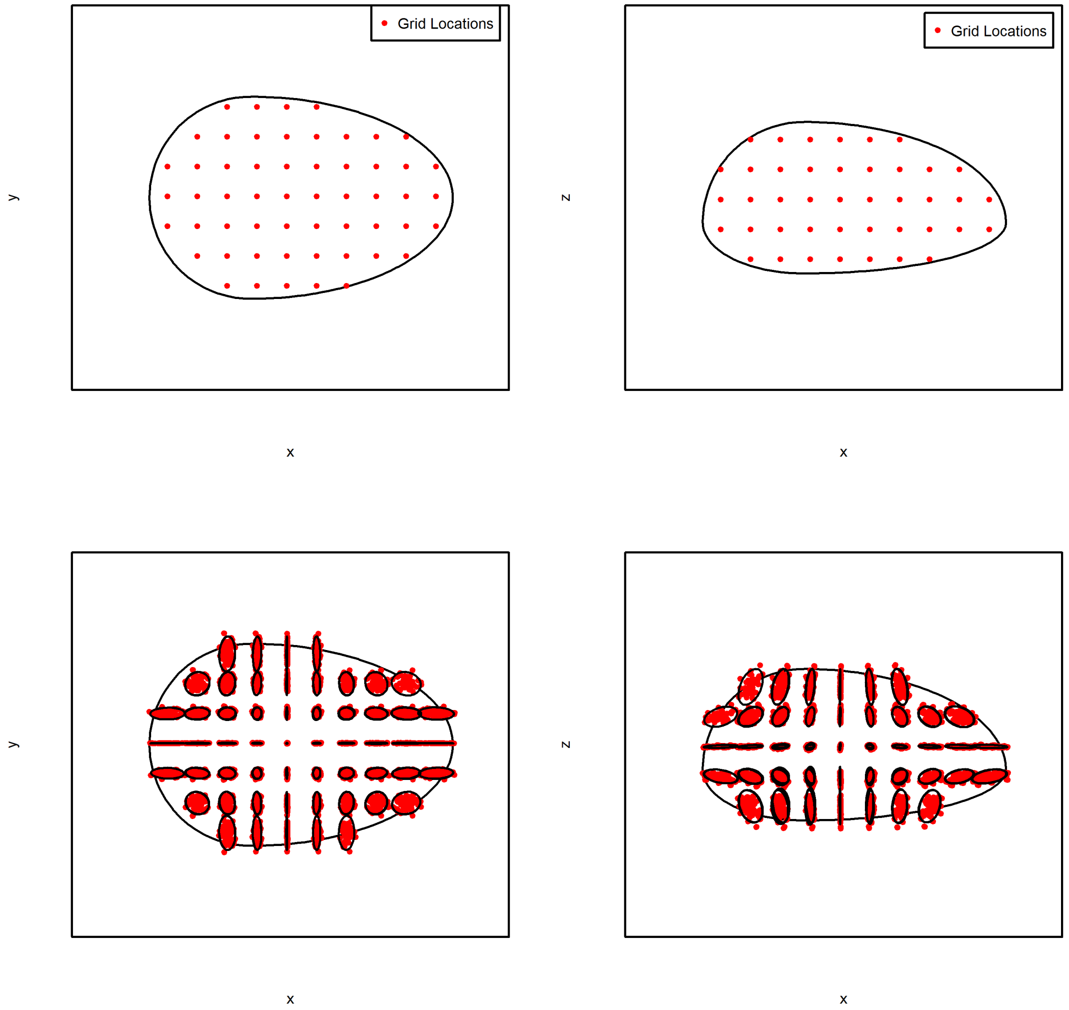

Figure 9: **The effect of thin-plate spline deformation using synthetic data.** The left column shows a 2D orthogonal projection in the x, y plane whereas the right column shows the same shape but projected into the x, z plane. The top row shows regularly spaced locations within the boundary of the shape. Fifty boundary shapes samples (the process for generating is described in the next section) are obtained where the regular locations are the same. The bottom row shows the TPS deformation of the regular locations across these 50 simulated images. The ellipses denote a contour that contain 95% of the locations mapped to this point, see text for details.

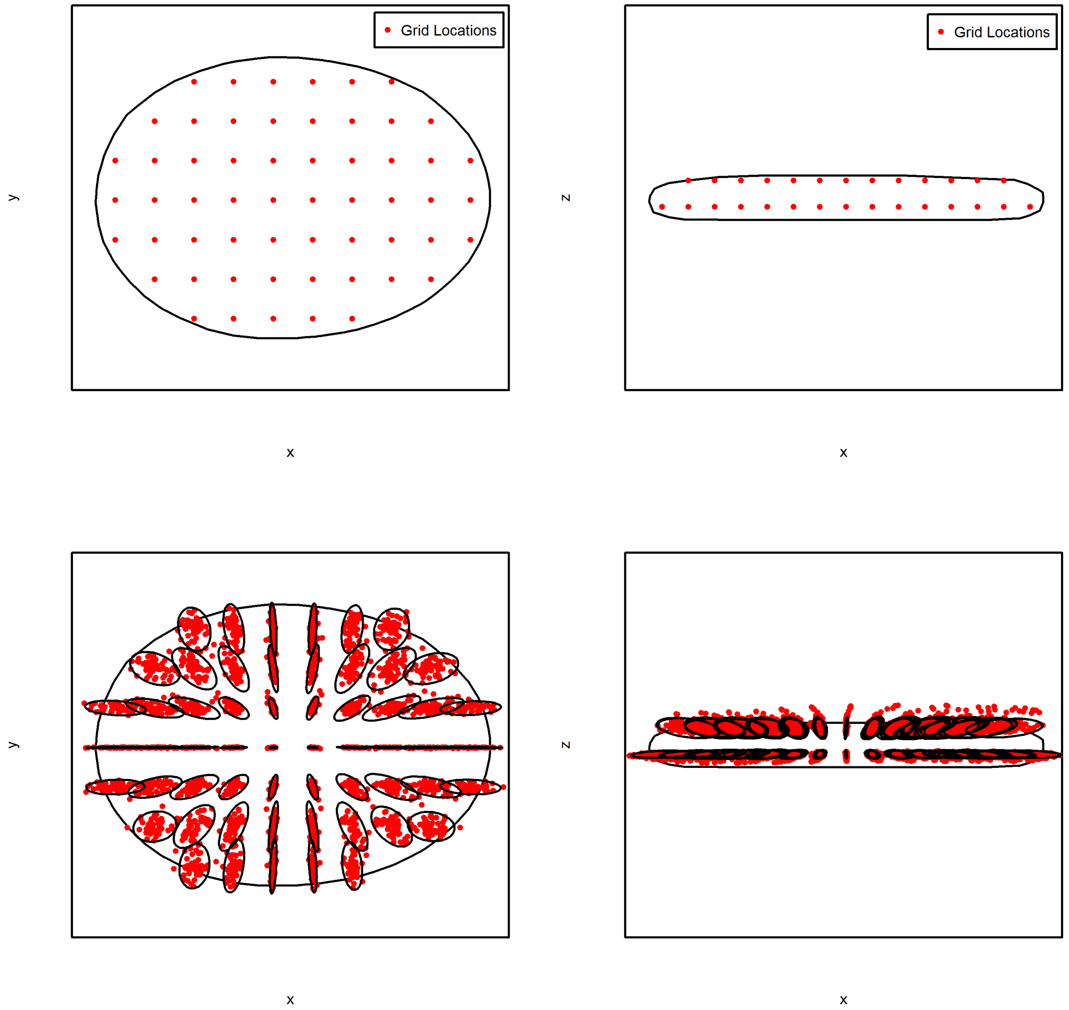

Figure 10: **The effect of thin-plate spline deformation**. This result use boundaries extracted from cell nuclei from the normal, asynchronous MRC-5 dataset. Details as Figure 9.

Mean shape

The synthetic nuclei boundaries generated to investigate the effect of the TPS transformation in the previous section can also be used to validate the process of mean shape estimation. In Figure 12, the ground truth shape is plotted along with mean shape, we can see the mean shape is a good estimation of the true shape. However it must be noted that this estimation was achieved using the correct orientation of the synthetic nuclei, which is dependent on identifying the location of the landmark for the ovoidal tip. This leads us to the issue of landmark identification which we discuss in the following section.

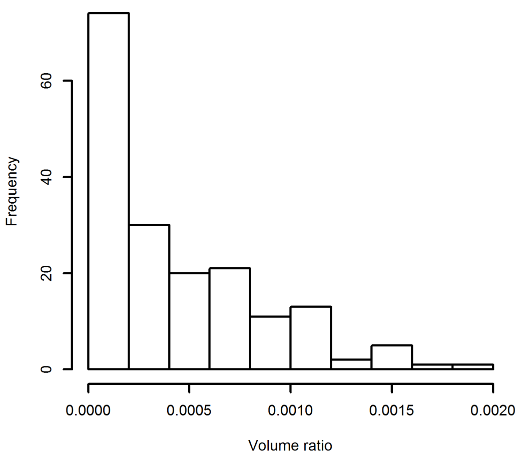

Figure 11: **Ratio of Volume of 95% Confidence Contour to volume contained within the shape boundary.** A) Synthetic shape set. The shape boundary is the true shape B) The MRC5 nuclei boundary set, where the shape boundary is the mean shape

Landmark identification issues

We now consider issues regarding the use of the ovoidal tip landmark. This landmark essentially adds a second preferred direction to the nuclei which is in the x-y plane. The existence of this direction and indeed the direction of gravity may not have any significance to the spatial point process underlying the spatial point patterns. In which case the alignment is arbitrary, however spatial point patterns that are invariant to rotation will still be recovered.

Aggregate Maps constructed from Synthetic Data

Here we consider the impact of the detection capability of the methodology against simulated data. The simulation engine is detailed in [8]. In summary, a synthetic boundary is constructed from a piece-wise function of ellipsoids to produce four different types of shape, as shown in Figure 3 from [8]. Fifty instances of each of these shapes are generated by perturbing the parameters that specify each shape. The scale of perturbations was chosen to produce shape boundaries that are in some sense similar to the boundaries from normal, synchronous MRC-5 dataset, see Figure 4 from [8]. The following experiment shows the result of combining all 4 different shape types from the 'thin' dataset into one AM. In these experiments we assume we know the correct location for the ovoidal tip landmark, so we can correctly align each synthetic cell nucleus. We consider 3 types of spatial point processes:

1. Polar, which has a preference for the ends of the longest axis within the x, y projection.

2. Center which has a preference for the center of the boundary.

3. Boundary which has a preference for the boundary in x, y projection.

The number of events in each realization is determined by sampling a Poisson distribution with intensity 32 (rejecting samples with less than 5 to avoid empty or sparsely populated cells). Each of

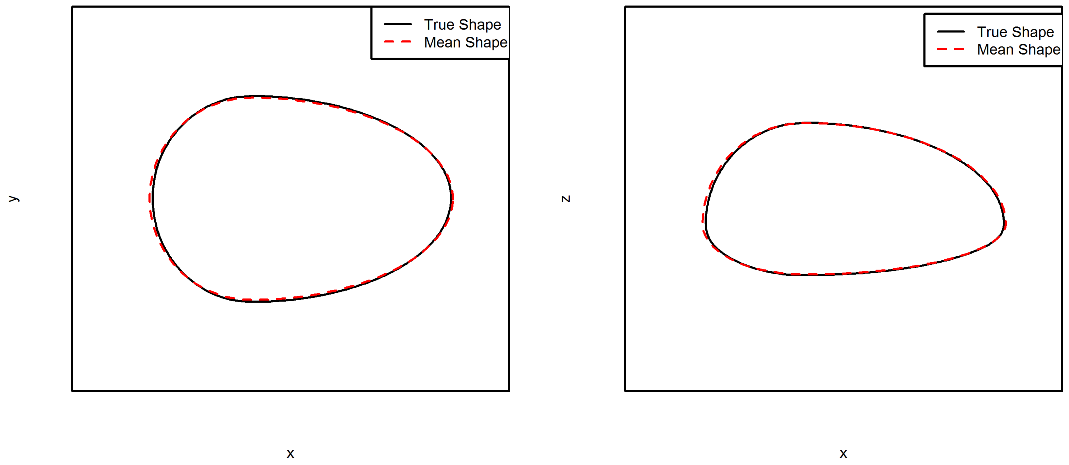

Figure 12: The ground truth shape for the nucleus that is perturbed to generate the synthetic data for Figure 9 and the estimated mean shape.

the spatial point processes is generated using thinning, as described in Materials and Methods Section of [8]. The Boundary processes in the following experiment refers to the distance to the boundary in a orthogonal projection onto the x, y plane.

Figure 14 shows AMs for the Center point process in which events are more likely found in the centre, this is clearly evident in this image. For both Polar and Boundary, Figures 13 and 15 respectively, the AMs suggest a strong rejection of regions where the events are more dispersed than is expected under CSR. However regions that are more aggregated are shown as being consistent with CSR. This disparity can be explained by the actual shape of the boundary. The issue here is that the size of clusters near the boundary are restricted in size, but are compared with CSR clusters that do not have this restriction. For this reason it is appropriate to show AMs for dispersion and aggregation together in order to get a better overall understanding of the underlying spatial point processes.

Finally we created synthetic nuclei that contained events following CSR. Figure 16 shows the scale of false positive clusters. In this case a false positive region that has been determined to be more dispersed than expected by CSR can be seen. The proportion of false positive voxels to the total number of voxels in the cell boundary is 0.1%.

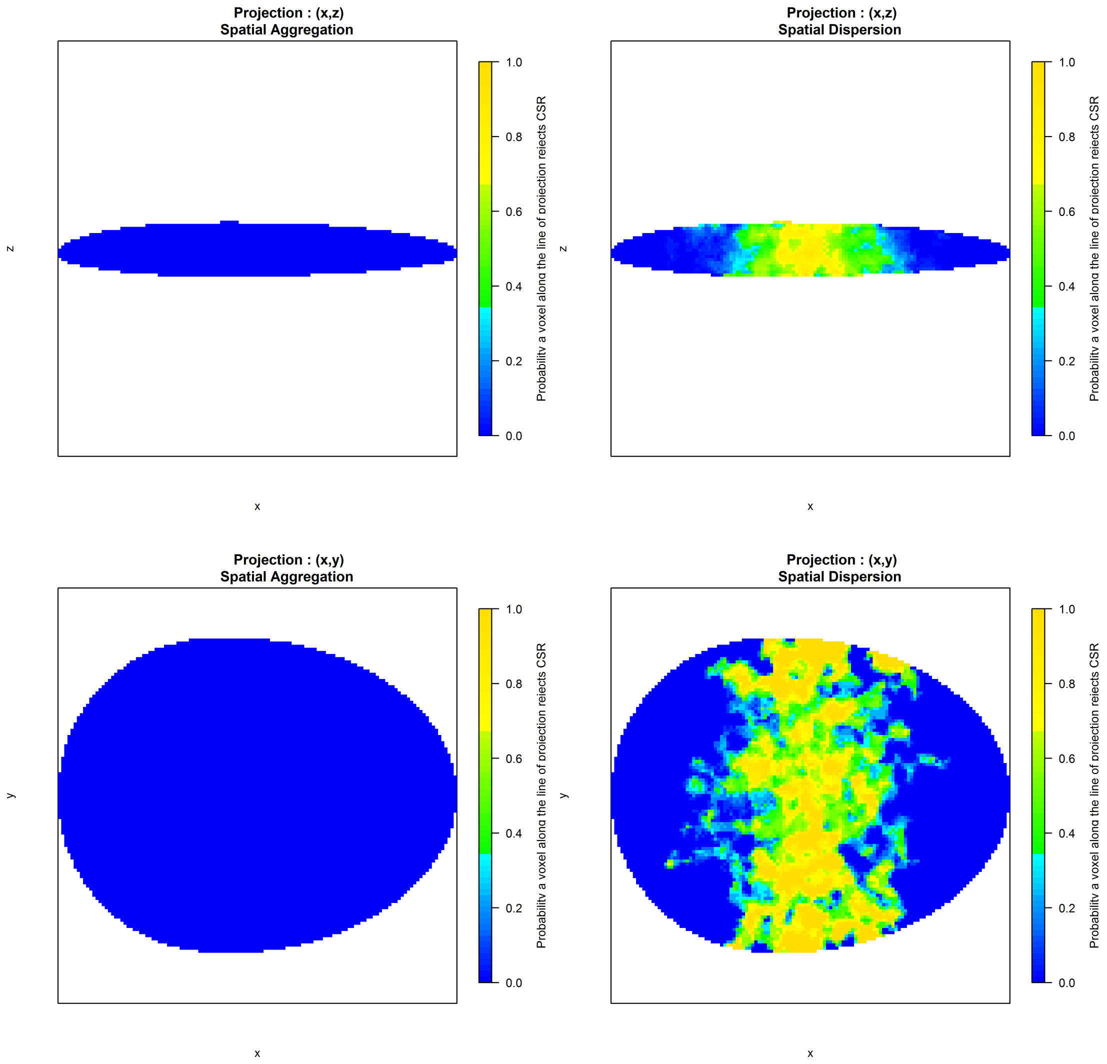

Figure 13: **The AM for Polar Spatial Point Process.** The top row shows the AM projected onto the x, z plane and the bottom row onto the x, y plane. The first column shows regions that are more aggregated than is expected through CSR, the second column shows regions that are more dispersed than CSR. Note that the AM fails to reject the null near the ends, where there are more points than would be expected under CSR. This is due to the TFCE cluster sizes being prevented from 'growing' any larger due to the boundary.

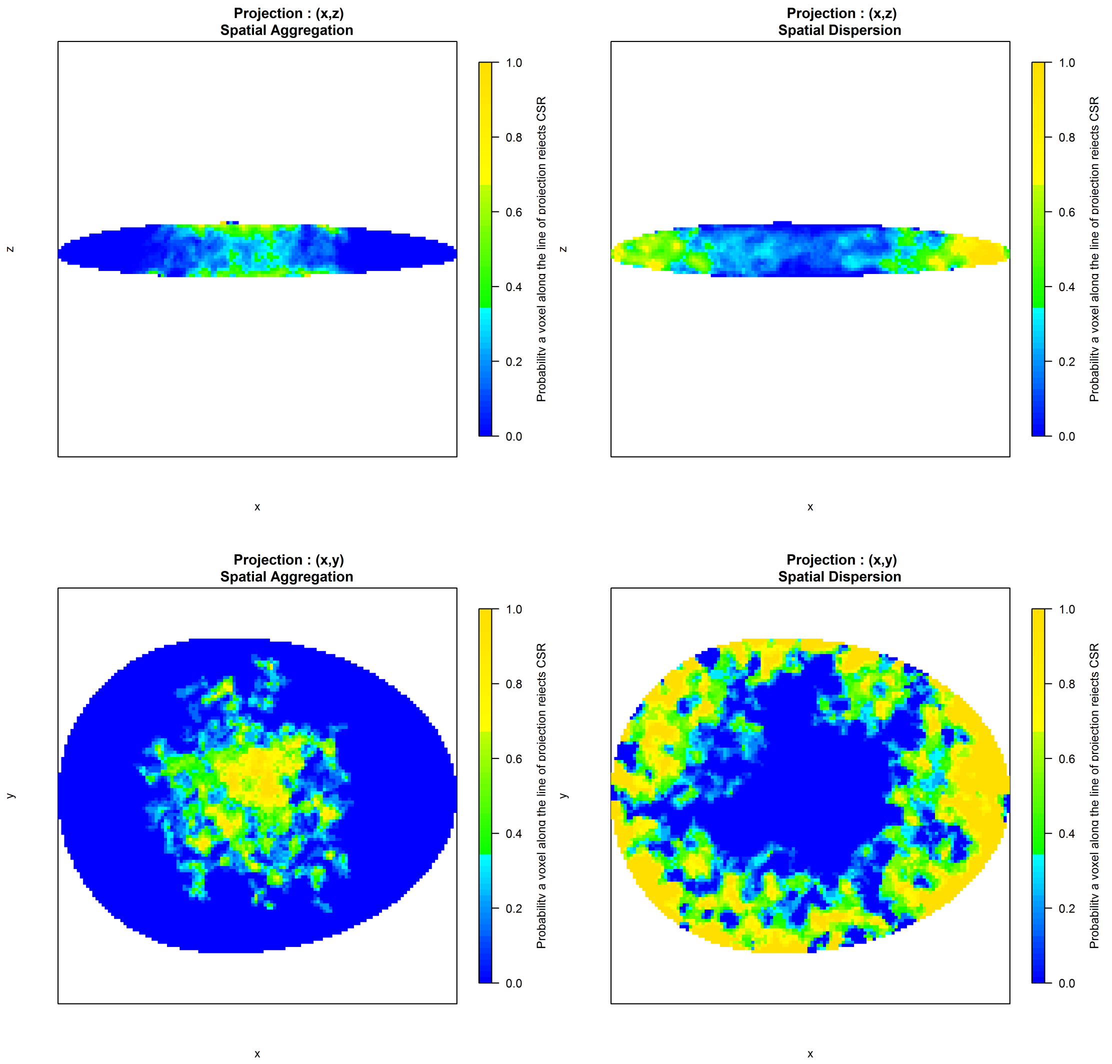

Figure 14: **The AM for Center Spatial Point Process.** The top row shows the AM projected onto the x, z plane and the bottom row onto the x, y plane. The first column shows regions that are more aggregated than is expected through CSR, the second column shows regions that are more dispersed than CSR.

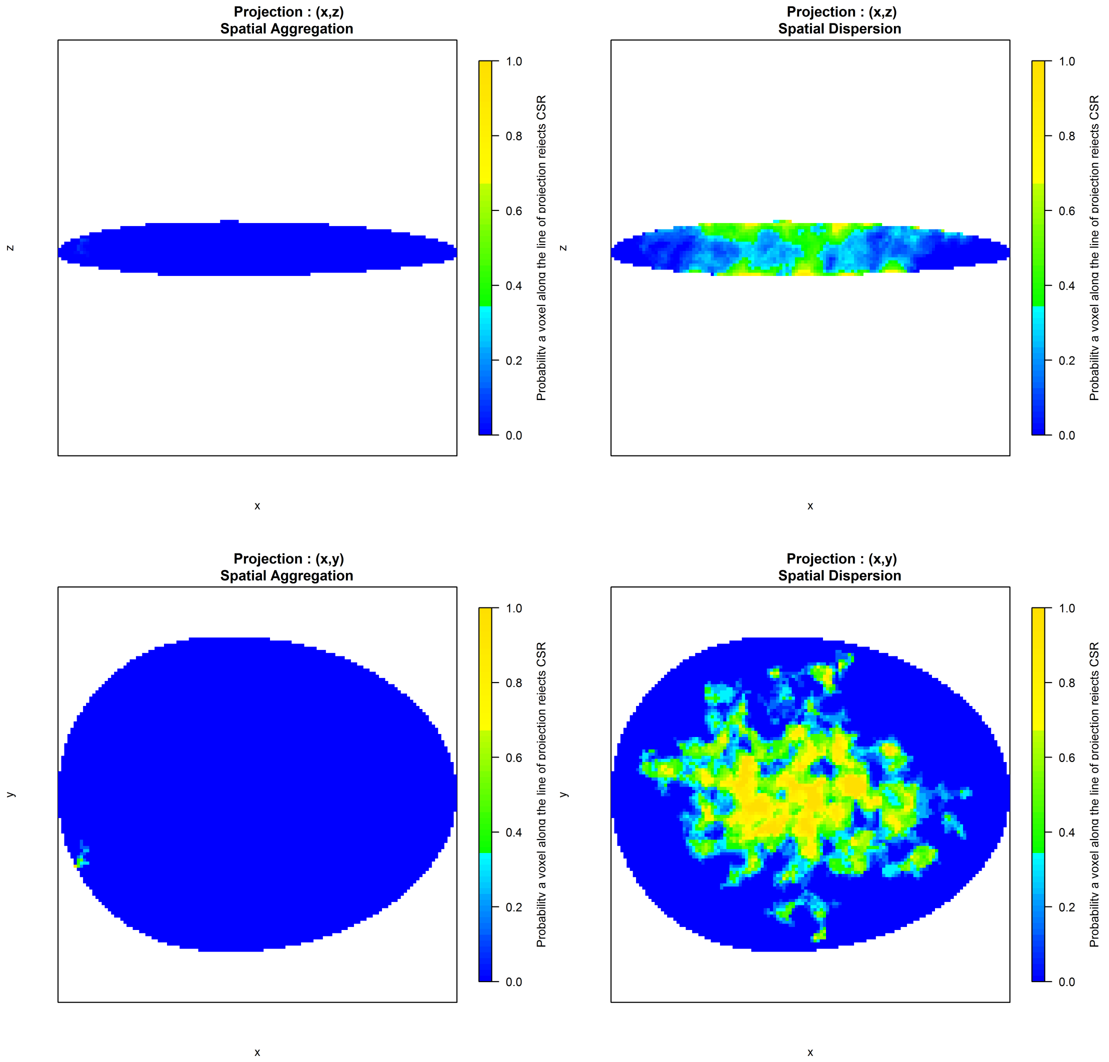

Figure 15: **The AM for the Boundary Spatial Point Process.** The top row shows the AM projected onto the x, z plane and the bottom row onto the x, y plane. The first column shows regions that are more aggregated than is expected through CSR, the second column shows regions that are more dispersed than CSR. Note that there are no voxels that are determined to be more aggregated than expected under CSR, this is due to the regions that are more aggregated are found close to the boundary. The boundary prevents the size of the cluster from becoming large enough to be able to reject the null hypothesis of CSR.

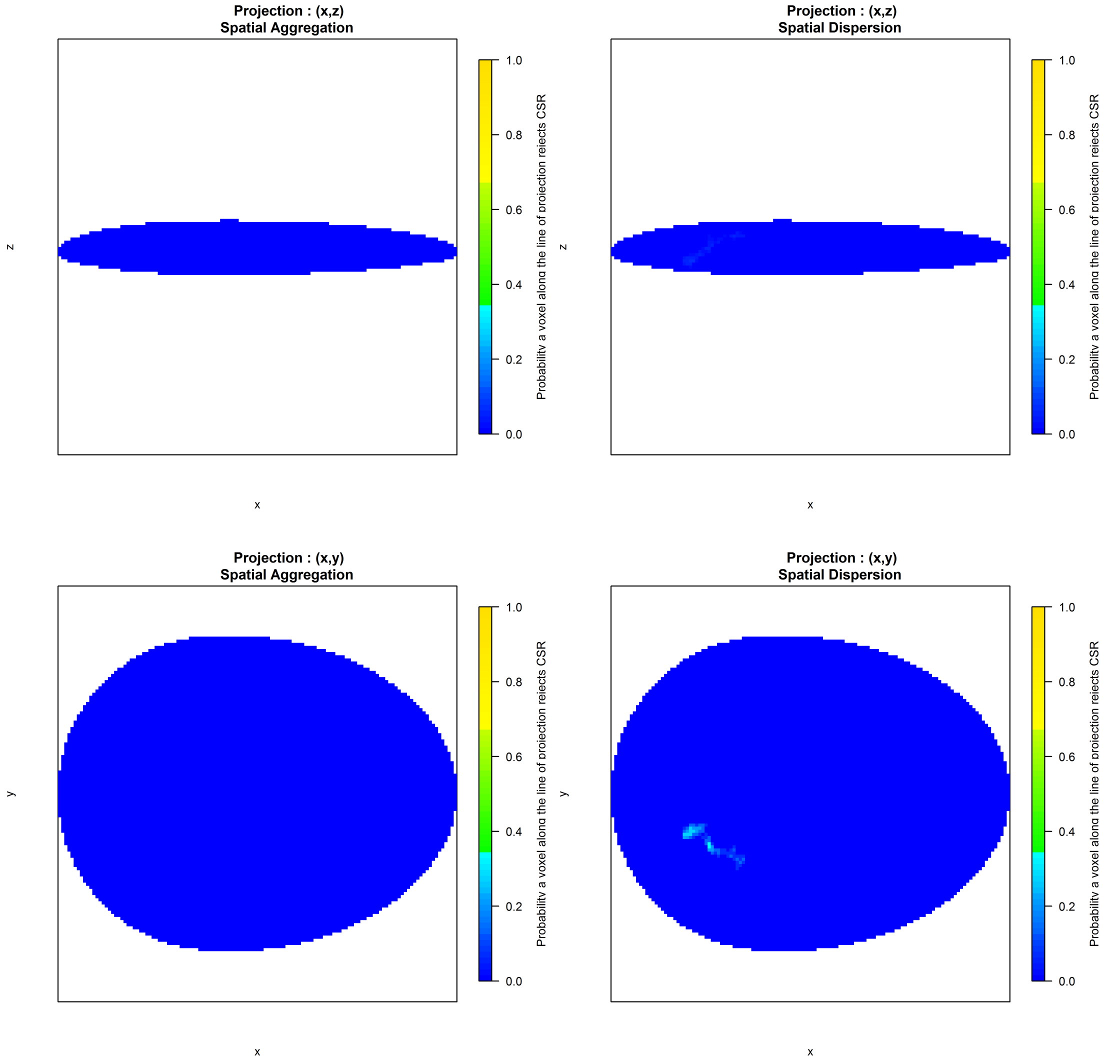

Figure 16: **The AM for the CSR Spatial Point Process.** The top row shows the AM projected onto the x, z plane and the bottom row onto the x, y plane. The first column shows regions that are more aggregated than is expected through CSR, the second column shows regions that are more dispersed than CSR. These images demonstrate the scale of false positives. The proportion of voxels that reject the null is 0.1%

References

1. Dryden I, Mardia K. Statistical Shape Analysis. Wiley; 1998.

2. Bier EA, Sloan KR. Two-part texture mappings. Computer Graphics and Applications, IEEE. 1986;6(9):40–53.

3. Braun J, Sambridge M. A numerical method for solving partial differential equations on highly irregular evolving grids. Nature. 1995;376:655 – 660.

4. Park SW, Linsen L, Kreylos O, Owens JD, Hamann B. Discrete Sibson interpolation. Visualization and Computer Graphics, IEEE Transactions on. 2006 March-April;12(2):243 –253.

5. Schaap W. DTFE: The Delaunay Tessellation Field Estimator. University of Groningen; 2007.

6. Regularized tessellation density estimation with bootstrap aggregation and complexity penalization. Pattern Recognition. 2012;45(4):1531 – 1539.

7. Threshold-free cluster enhancement: Addressing problems of smoothing, threshold dependence and localisation in cluster inference. NeuroImage. 2009;44(1):83 – 98.

8. Weston DJ, Adams NM, Russell RA, Stephens DA, Freemont PS. Analysis of Spatial Point Patterns in Nuclear Biology. PloS One. 2012;7(5):e36841.
